# Effect of Plant Spacing on Yield and Yield Components of Tomato (*Solanum lycopersicum* L.) in Shewarobit, Central Ethiopia

**DOI:** 10.1155/2020/8357237

**Published:** 2020-09-12

**Authors:** Getachew Amare, Hailay Gebremedhin

**Affiliations:** ^1^Department of Horticulture, College of Agriculture and Natural Resources Sciences, Debre Berhan University, P.O. Box: 445, Debre Berhan, Ethiopia; ^2^Department of Horticulture, College of Agriculture and Environmental Sciences, Debre Berhan University, P.O. Box: 50, Debre Berhan, Ethiopia

## Abstract

Inappropriate spacing is one of the major problems in tomato production at the study area. A field experiment was conducted to determine inter- and intrarow plant spacing for yield and yield components of tomato at Shewarobit, central rift valley of Ethiopia, under irrigation condition. The treatment comprises of three intrarow spacing (20, 30, and 40 cm) and four interrow spacing (60, 80, 100, and 120 cm) replicated three times and arranged in randomized complete block design using tomato variety Weyno. Data collected on fruit yield and yield components were analysed using SAS. The main effect of interrow spacing significantly affected marketable fruit, unmarketable fruit, marketable fruit number, unmarketable fruit number, and fruit diameter. The 20 cm interrow spacing showed a marked increase in marketable fruit yield by 35.96% as compared to 30 cm spacing used by farmers. Planting tomato in closer interrow spacing (60 cm) resulted in 50% yield increment than the widest (120 cm) space between rows. Interaction effects of both inter- and intrarow spacing significantly (*p* < 0.05) affected plant height and fruit length. An intrarow and interrow spacing of 20 ∗ 100 cm and 20 ∗ 120 cm resulted in tallest plants and widest fruits, respectively. Therefore, farmers can use 20 cm intrarow spacing and 60 cm interrow spacing for planting of tomato seedling of Weyno variety.

## 1. Introduction

Tomato (*Solanum lycopersicum* L.) belongs to the *Solanaceae* family, and it is amongst the main important and popular vegetables. The centre of origin for tomato assumed to be the Andean zone. It is reported that tomato was domesticated in Mexico and that the name of tomato was derived from the “tomatil” in the Nahua tongue of Mexico [1]. It is a good source of vitamins and minerals as it supplies Vitamin A, B, C, and D, minerals, Ca, P, and Fe [2]. One medium ripe tomato (∼145 grams) can provide up to 40% of the Recommended Daily Allowance of vitamin C and 20% of vitamin A [3].

It ranks next to potato in the area of cultivation, and it is the first as a processing crop among vegetables [4]. It is extensively produced by small-scale farmers and commercial producers and widely consumed fresh or processed. In Ethiopia, tomato is the main source of income and food security in most rift valley areas. According to Gemechis et al. [5], it is consumed in every household in different modes, but in certain areas, such as Wallo, Hararge, Shewa, Jimma, and Wallaga, acts as important costaple food. Desalegne [6] reported that many varieties of tomato including fresh and processed varieties are popular and economically important vegetable crop in small and large-scale farming.

Apart from the importance and significant effort made, the national average yield of tomato in Ethiopia is 5.3 tons ha^−1^ [7]. The average production is incomparable with the average yield of other countries such as China (59.4 tons ha^−1^), India (24.6 tons ha^−1^), USA (96.8 tons ha^−1^), Turkey (68.8 tons ha^−1^), and Egypt (40.9 tons ha^−1^) [8].

This low level of production is associated with many problems in relation with poor agronomic management practices such as planting density, fertilizer, and irrigation water management. Plant spacing is one of the agronomic factors which significantly affect tomato production. Optimum plant spacing enhances better utilization of spaces, high yield, and quality production [1]. Abrha et al. [9] reported that the interaction effect of inter- and intrarow spacing significantly affects both marketable and unmarketable yield of tomato. In Ethiopia, 10 to 50 cm intrarow spacing and 60 to 120 cm interrow row spacing were reported for different tomato varieties in different location and years. Lemma [10] underlined the importance of recommending inputs and management practices are central to produce a good yield and quality of tomato fruit depending on the varieties and growing region. In the study area, little information is available on plant spacing of the newly released semi-indeterminate variety of tomato called “Weyno.” This is one of the factors which lead to low yield and less quality production. Hence, the objective of this study was to evaluate the effects of plant spacing on fruit yield and associated growth characteristics of the Weyno variety of tomato at Shewarobit, central rift valley areas of Ethiopia.

## 2. Materials and Method

### 2.1. Description of the Study Area

**Figure 1:**
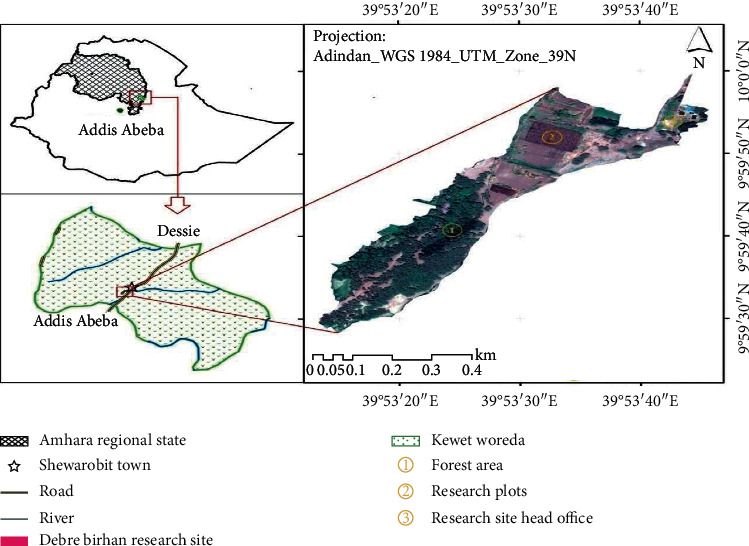
Map of the study area.

The experiment was conducted at Shewarobit, Kewet (Figure 1) district of North Shewa zone, Amhara national regional state. It is found at about 225 km northeast of Addis Ababa. It is located at 11° 55′ N latitude and 37° 20′ E longitude at an altitude of 1380 m.a.s.l (meters above sea level). It has an average annual rainfall of 1007 mm, with short rain between March and April and long rain between June and September and annual mean minimum and maximum temperatures of 16.5 and 31°C, respectively [11]. This research was conducted in the Debre Berhan University research and demonstration site which is classified as vertosol.

### 2.2. Experimental Treatment, Design, and Procedure

The experimental material used in this experiment was tomato using variety Weyno (semi-indeterminate type). Intrarows spacing of 20, 30, and 40 cm and inter-row spacing of 60, 80, 100, and 120 cm were set in randomized complete block design with factorial scheme. Each treatment was duplicated three times making 36 experimental units with a treatment combination of 12. The spacing between plots and blocks was 75 cm and 1 m, respectively.

The land was tilled to a depth of 35 cm, and the nursery bed was neatly prepared. The seeds were collected from the Srinka Agriculture Research Centre where the Weyno variety is released and maintained. The seeds were separately drilled into the beds, covered lightly with a film of loosed soil, and mulched immediately using dry grasses. After emergence, the mulch material was removed to harden the seedling. The tomato seedlings were transplanted four weeks after planting into a well-harrowed and ridged field.

### 2.3. Data Collected

Data were collected on the following:

  Plant height (cm): the plant height was measured from the base of the plants to the terminal growing point of the main stem at the end of the third harvest. The average plant height of 10 randomly selected plants per plot was measured and expressed in centimetres (cm).
  Number of primary branches per plant: the number of primary branches of randomly selected 10 plants from each plot was recorded.
  Days to 50% flowering: when the flowering was noticed in the 50% of the plants per plot, it was considered as 50 percent flowering and days taken to this stage were considered as days to 50 percent flowering and expressed in number.
  Marketable fruit number per hectare: those fruits from the ten tagged plants, which were free from visible damage, insect pest, diseases and not extra small-sized (>20 g), were considered as marketable. The fruits were counted at each harvest time, averaged converted to number of marketable fruits per hectare, and expressed in numbers
  Unmarketable fruit number per hectare: fruits with cracks, rotting, damage by insects, diseases, and birds, and sunburn, as well as extra small-sized fruits, were collected from ten tagged plants and were considered as unmarketable. The fruits were counted at each harvest time and expressed in numbers and converted to number of unmarketable fruits per hectare.
  Marketable fruit weight per hectare (kg ha^−1^): the fruits harvested from two middle rows, which were free from damage by insect pests and diseases and not extra small-sized (>20 g), were weighed, averaged, and expressed in yield per plot and calculated on per hectare basis.
  Unmarketable fruit weight per plant (kg ha^−1^): fruits with cracks, rotting, damage by insects, diseases, and birds, and sunburn, as well as extra small-sized fruits (<20 g) which were collected from the two middle rows, were considered as unmarketable and, then, converted to per hectare basis.
  Fruit length (cm): the fruit length of 10 randomly selected marketable fruits from each plot at each harvest was measured using a caliper meter, and their average is expressed as fruit length.
  Fruit diameter (cm): the diameter of 10 randomly selected fruits per each plot at each harvest was measured using a caliper meter, and their average is expressed as fruit diameter.

### 2.4. Data Analysis

The data were subjected to Analysis of Variance (ANOVA) using the Statistical Analysis System (SAS), version 9.2 (2009). Detection of differences among treatment means for significance was performed using DMRT (Duncan's multiple range tests at 0.05 probability level).

## 3. Results and Discussion

### 3.1. Marketable Fruit Yield

Marketable fruit yield was significantly (*p* < 0.05) affected by inter- and intrarow spacing, whereas the interaction effect of intra- and interrow spacing was not significant (Table 1). The highest marketable fruit yield was recorded at closer intra- and interrow spacing (Table 2). Planting with an intrarow spacing of 20 cm resulted in a higher yield of 35.96% and 36.97% compared to 30 and 40 cm planting distance, respectively. In the case of the furrow distance, the highest marketable fruit yield was recorded from 60 cm interrow spacing (7674 kg), which is statistically in par with 80 cm. However, planting arrangement of 100 and 120 cm resulted in a reduced yield of 49.85% and 50.21% compared to 60 cm, respectively. These results clearly indicated that farmers may lose much more yield if they do not use appropriate planting distance.

The reason for the higher marketable fruit yield in the narrow interrow and intrarow spacing could be attributed due to more plant populations per unit area. A higher number of plants is related to a higher number of fruit clusters and a higher number of fruits and, thereby, higher yield [12, 13]. On the other hand, when the number of plants per unit area decreased, the yield also decreased. Similarly, other findings confirm the highest marketable yield obtained from closer plant spacing [14, 15]. In contrast with the current result, other findings indicated that higher marketable yield was recorded from wider interrow spacing than closer spacing [16, 17]. These contrasting results might be due to variations in crop management practices, variety, and environmental conditions where the crop is grown.

### 3.2. Number of Marketable and Unmarketable Fruit

As shown in Table 1, interrow spacing significantly (*p* < 0.05) affected the number of marketable and unmarketable fruit yield, whereas the main effect of intrarow spacing and its interaction with interrow was not significant (*p* > 0.05). The highest number of unmarketable and marketable fruit was recorded from a closer spacing of 60 cm followed by 80 cm interrow spacing with no significant difference in between. This could be due to high plant population per unit area. The high number of plants per unit area increased flower clusters which increased number of fruits. This result is in line with that of Maboko et al. [16] who indicated that higher number of marketable fruits is obtained on closer spacing. In contrast to this result, other researchers reported that higher number of marketable and unmarketable fruits was obtained in wider plant spacing [4, 17]. According to their report, high number of marketable and unmarketable fruit yield per plot was attributed to having less computation for water and nutrients in wider spacing than in closer ones.

### 3.3. Unmarketable Fruit Yield

The unmarketable fruit yield per hectare was significantly (*p* < 0.05) affected by interrow spacing. The highest unmarketable fruit yield was recorded from 60 cm interrow spacing (3961 kg) but statistically similar with 80 cm interrow spacing (3701.5 kg). On the other hand, the lowest unmarketable fruit yield was recorded from 120 cm inter- and 40 cm interrow spacing, which was statistically at par with 100 cm interrow spacing. High unmarketable fruit yield was recorded from closer spacing. This might be due to higher inter- and intrarow competition for resources such as light, water, and nutrients. Such stiff competition for nutrients and minerals could cause production of small-sized fruits, cracked fruits, damaged fruits by insects, disease, and birds, and sun burn. On the other hand, this result disagrees with Ara et al. [1] who reported that the highest unmarketable fruits were recorded from wider spacing rather than closer spacing. This could be due to variability in the soil type and differences in the growing environment. Conversely, Papadopoulos and Ormrod [18] found low proportion of unmarketable fruit grade of tomato under greenhouse condition which is grown in wider spacing. Maboko et al. [16] and Desalegne [6] also reported that the closer the spacing between rows, the minimum was the number of sun-scorched fruits. This clearly indicated that plant spacing should be determined by considering cultivar, site, and management practices required.

### 3.4. Fruit Diameter

Fruit diameter was significantly (*p* < 0.05) affected (*p* < 0.01) by the main effect of interrow and intrarow spacing (Table 1), whereas the interaction effect of intra- and interrow spacing was not significant. The highest fruit diameter (5.7 cm) was observed by an interrow spacing of 100 cm, and the lowest fruit diameter was recorded by 60 cm interrow spacing. Intrarow spacing 30 cm recorded higher fruit diameter and the lowest was from an intrarow spacing of 20 cm. The highest fruit diameters from wider inter- and intrarow spacing than closer ones will be due to less competition for light and nutrients. Hence, it may produce large-sized fruits, as there will be sufficient light and available nutrients for plants. This result is in agreement with Asefa et al. [19] who reported that higher fruit diameter was recorded when the intrarow spacing increased from 20 to 40 cm and from 70 cm to 100 cm interrow spacing. On the other hand, in contrast with this result, Chernet et al. [20] reported that fruit diameter was not significantly affected by inter- and intrarow spacing.

### 3.5. Number of Primary Branches

Results indicated that intrarow and interrow spacing had a nonsignificant (*p* > 0.05) effect on the number of primary branches (Table 1). Branch and reproductive structures are more dependent on cell division, which are mostly determined by the genetic characteristics of the plant. However, planting distance had shown no remarkable effect on primary branches of the tomato variety “Weyno” in the current experiment. This could be due to the genetic nature of the plant which is much responsive for yield and yield-related components (Table 2) rather than ontogeny and phenological characteristics of the plant.

### 3.6. Days to 50% Flowering

Intrarow spacing and interrow spacing had shown nonsignificant (*p* > 0.05) effects on days to 50% flowering of tomato under Shewarobit condition (Table 2). In many cases, the reproductive cycle of crops is much dependent of their genetic makeup rather than management practices. This could be the reason for the nonsignificant result obtained in this planting distance. Opposite to these results, Agele et al. [21] reported that the onset of flowering and 50% flowering date were significantly earlier as plant density decreased.

### 3.7. Plant Height

Significant (*p* < 0.01) differences was observed in plant height due to the main effects of inter- and intrarow spacing. Similarly, interaction effect of inter- and intrarow spacing significantly affected plant height (Table 2). The tallest (112 cm) and shortest (73 cm) plant height found in plants grown in 20 × 100 cm and 20 × 60 cm planting distance, respectively (Table 2). This could be due to the increased competition between plants within the row and reduced competition between rows, which favours proportional growth and in, then, good plant height. The current result was in accord with the results of Chernet et al. [20] who reported that the widest interrow spacing of 100 cm and closer spacing of 20 and 30 cm intrarow spacing gave the tallest plant. Ara et al. [1] also reported that the tallest plant height was recorded from wider spacing 40 cm intrarow spacing than 30 cm spacing. In the contrary, different researchers reported higher plant height in closer spacing than wider spacing [17, 22, 23].

### 3.8. Fruit Length

Interaction effect of inter- and intrarow spacing significantly (*p* < 0.05) affected fruit length. But, main effects were not significantly affected by inter- and intrarow spacing (Table 1). The highest fruit length (7.63 cm) was recorded from the interaction of 20 × 120 cm intra- and interrow spacing, but statistically at par with other spacing interactions except 120 × 40 cm. An intra- and interrow spacing interaction 20 × 60 cm gave the shortest (6.03 cm) fruit length (Table 2).

Enhanced fruit length in interaction of closer intrarow spacing (20 cm) and wider inter-row spacing (120 cm) could be because of reduced interrow competition for resources such as radiation, fertilizer, and moisture. On the other hand, shortest fruit length recorded from the interaction of closer spacing of 20 × 60 cm could be due to the impact of competition for nutrients and water in closer spacing, which favours the formation of short fruits as the nutrient is not optimum enough to give good fruit length. The result also indicated that all interrow spacing interacted with 30 cm intrarow spacing do not show significant difference in fruit length. This could verify that this intrarow spacing is from other interrow spacing treatments. In agreement with this research, Abrha et al. [9] reported that the shortest fruit length was recorded from a closer intrarow spacing of 20 cm. Ogundare et al. [23] also reported that highest fruit length was recorded from a wider spacing of 75 × 60 cm than closer spacing.

## 4. Conclusions

Generally, this research result showed that an intrarow spacing of 20 cm increased marketable fruit yield and an interrow spacing of 60 cm resulted higher marketable fruit yield, unmarketable fruit yield, and the number of marketable and unmarketable fruit yield. The interaction of intra- and interrow spacing of 20 × 120 cm resulted in higher plant height. Similarly, 20 × 100 resulted in higher fruit length with no significant difference to other interaction effects of inter- and intrarow spacing. Regardless of interrow spacing, using 20 cm intrarow spacing increased marketable fruit yield by 35.96% as compared to the frequently used spacing by farmers which is 30 cm. More than 50% yield variation was also obtained when closer interrow spacing (60 cm) was used compared to the widest (120 cm) space between rows. Therefore, farmers can use 20 cm intrarow spacing and 60 cm interrow spacing for higher yield and quality fruit production of the Weyno variety.

## Figures and Tables

**Table 1:** Main effect of intrarow spacing on yield and yield components of tomato at Shewarobit

		MFY (kg ha^−1^)	UMFY (kg ha^−1^)	NMF	NUMF	FD (cm)	No. branch	DTF (days)
*Intra‐row spacing*							
20 cm		6992^a^	756.56	155525	138.1	4.78^b^	7.67	44.08
30 cm		4477^b^	858.80	110747	148.8	5.39^a^	8.17	44.33
40 cm		4419^b^	892.31	98239	122.8	5.05^ab^	7.00	44.33
Sig. level		*∗*	ns	ns	ns	*∗*	ns	ns
CV (%)		49	51	54	54	10.8	33.8	9.4

*Inter‐row spacing*							
60 cm		7674^a^	3961.0^a^	207529^a^	198.3^a^	4.5189^c^	44.8	7.1
80 cm		5840^ab^	3701.5^ab^	122558^ab^	153.2^ab^	4.8733^bc^	44.7	6.6
100 cm		3848^b^	2024.9^b^	83003^b^	103.7^b^	5.7067^a^	43.9	7
120 cm		3821^b^	1749.4^b^	72924^b^	91.1^b^	5.0589^b^	43.7	6.9
Sign. level		*∗*	*∗*	*∗*	*∗*	*∗∗*	ns	ns

CV (%)		49	51	54	54	10.8	9.4	7.8

Means in columns with the same letter(s) in each treatment are not significantly different, UMF: unmarketable fruit yield; NMF: number of marketable fruit; NUMF: number of unmarketable fruit yield; FD: fruit diameter; No. branch: number of primary branches; PH = plant height; FL = fruit length; DTF: days to first flowering.

**Table 2:** Interaction effect of inter- and intrarow spacing on plant height and fruit length of tomato.

Interrow spacing (cm)	Intrarow spacing (cm)					
	Plant height			Fruit length		
	20	30	40	20	30	40
60	73^e^	82^d^	85^d^	6.03^c^	6.70^abc^	7.13^ab^
80	100.3^bc^	103.6^b^	101.3^bc^	7.43^ab^	6.87^abc^	7.00^abc^
100	112^a^	104^b^	105^b^	7.47^ab^	6.87^abc^	6.70^abc^
120	95.6^c^	94.6^c^	96^c^	7.63^a^	6.90^abc^	6.43^bc^
Sign. level		*∗∗*			*∗*			
CV (%)		16.4			7.8	

Means in columns and row with the same letter(s) within each plant parameter are not significantly different.

## Data Availability

Data are available on request from Getchew Amare and Hailay G/medhin (e-mail: getchamare38@gmail.com).

## References

[1] Ara N., Bashar M., Begum S., Kakon S. (2007). Effect of spacing and stem pruning on the growth and yield of tomato. *International Journal of Sustainable Crop Production*.

[2] Jones J. B. (2007). *Tomato Plant Culture: In the Field, Greenhouse, and Home Garden*.

[3] Kelley W. T., Boyhan G. (2010). *Commercial Tomato Production Hand Book*.

[4] Muhammad A., Singh A. (2007). Intra-row spacing and pruning effects on fresh tomato yield in Sudan Savanna of Nigeria. *Journal of Plant Sciences*.

[5] Gemechis A. O., Struik P. C., Emana B. (2012). Tomato production in Ethiopia: constraints and opportunities. http://www.tropentag.de/2012/abstracts/full/659.pdf.

[6] Desalegne L. (2002). *Tomatoes Research Experiences and Production Prospects*.

[7] CSA (Central Statistical Agency) (2018). *Key Findings of the 2018. Agricultural Sample Surveys, the Federal Democratic Republic of Ethiopia*.

[8] FAOSTAT (2018). The food and agriculture organization of the United Nations statistical database: romen farming. http://www.fao.org/faostat/en/#data/QC/visualize.

[9] Abrha H., Birhanu A., Desta M., Kebede A. (2015). Effect of inter and intra-row spacing on yield and yield components of tomato (*Solanum lycopersicum* linn.) in SouthTigray, Ethiopia. *Journal of Natural Sciences Research*.

[10] Lemma D. (2004). *Tomatoes: Research Experiences and Production Prospects, Research Report No. 43*.

[11] BoA (Bureau of Agriculture) (2000). *Kewet Woreda Agriculture Development Office, Annual Report*.

[12] Balemi T. (2008). Response of tomato cultivars differing in growth habit to nitrogen and phosphorus fertilizers and spacing on vertisol in Ethiopia. *Acta Agriculturae Slovenica*.

[13] Kirimi J. K., Itulya F. M., Mwaja V. N. (2011). Effects of nitrogen and spacing on fruit yield of tomato. *African Journal of Horticultural Science*.

[14] Law-Ogbomo K. E., Remison S. U. (2007). The response of Dioscorea rotundata to NPK fertilizer in Edo State, Nigeria. *Research Journal of Agriculture and Biological Sciences*.

[15] Getahun D., Bikis D. (2015). Responses of tomato varieties to intra-row spacing under rain-fed production. *Agricultural Science Research Journal*.

[16] Maboko M. M., Du Plooy C. P., Chiloane S. (2011). Effect of plant population, fruit and stem pruning on yield and quality of hydroponically grown tomato. *African Journal of Agricultural Research*.

[17] Hussen S., Kemal M., Wasie M. (2013). Effect of intra-row spacing on growth and development of tomato (*Lycopersicum esculentum* mill) var. Roma VF, at the experimental site of Wollo University, South Wollo, Ethiopia. *International Journal of Sciences: Basic and Applied Research (IJSBAR)*.

[18] Papadopoulos A. P., Ormrod D. P. (1990). Plant spacing effects on yield of the greenhouse tomato. *Canadian Journal of Plant Science*.

[19] Assefa W., Tesfaye B., Dessalegn L. (2015). Influence of inter and intra-rows spacing on yield and yield components of tomato cultivars. *Ethiopian Journal of Agricultural Sciences*.

[20] Chernet S., Belay F., Tekle G., Kahsay Y., Weldu N., Zerabruk G. (2017). Response of yield and yield components of tomato (Solanum Lycopersicon L.) to different inter and intra-row spacing at Merebleke, Northern Ethiopia. *African Journal of Agricultural Research*.

[21] Agele S. O., Iremiren G. O., Ojeniyi S. O. (1999). Effects of plant density and mulching on the performance of late-season tomato (Lycopersicon esculentum) in southern Nigeria. *The Journal of Agricultural Science*.

[22] Singh P., Singh K., Yadav C., Ganga J. (2012). Effect of intra-row spacing and pruning on yield, water use and economics of tomato production under naturally ventilated bamboo polyhouse. *Pantnagar Journal of Research*.

[23] Ogundare S. K., Oloniruha J. A., Ayodele F. G., Bello I. A. (2015). Effect of different spacing and urea application rates on fruit nutrient composition, growth and yield of tomato in derived savannah vegetation of Kogi state, Nigeria. *American Journal of Plant Sciences*.

