# Corrigendum to “Effect of Plant Spacing on Yield and Yield Components of Tomato (*Solanum lycopersicum* L.) in Shewarobit, Central Ethiopia”

**DOI:** 10.1155/2020/5395740

**Published:** 2020-11-22

**Authors:** Getachew Amare, Hailay Gebremedhin

**Affiliations:** ^1^Department of Horticulture, College of Agriculture and Natural Resources Sciences, Debre Berhan University, P.O. Box: 445, Debre Berhan, Ethiopia; ^2^Department of Horticulture, College of Agriculture and Environmental Sciences, Adigirat University, P.O. Box: 50, Adigirat, Ethiopia

In the article titled “Effect of Plant Spacing on Yield and Yield Components of Tomato (*Solanum lycopersicum* L.) in Shewarobit, Central Ethiopia” [1], author Hailay Gebremedhin was affiliated to “Department of Horticulture, College of Agriculture and Environmental Sciences, Debre Berhan University, P.O. Box: 50, Debre Berhan, Ethiopia,” which is incorrect. The correct affiliations for this author are as follows:

^2^Department of Horticulture, College of Agriculture and Environmental Sciences, Adigirat University, P.O. Box: 50, Adigirat, Ethiopia.

The corrected list of affiliations is shown in the author information above.
